# Discovery of hemocompatible bacterial biofilm-resistant copolymers

**DOI:** 10.1016/j.biomaterials.2020.120312

**Published:** 2020-11

**Authors:** Taranjit Singh, Andrew L. Hook, Jeni Luckett, Manfred F. Maitz, Claudia Sperling, Carsten Werner, Martyn C. Davies, Derek J. Irvine, Paul Williams, Morgan R. Alexander

**Affiliations:** a School of Pharmacy, University of Nottingham, Nottingham, NG7 2RD, UK; b Leibniz Institute of Polymer Research Dresden, Max Bergmann Centre for Biomaterials Dresden, Hohe Str. 6, D-01069, Dresden, Germany; c Department of Chemical and Environmental Engineering, Faculty of Engineering, University of Nottingham, Nottingham, NG7 2RD, UK; d Biodiscovery Institute and School of Life Sciences, University of Nottingham, Nottingham, NG7 2RD, UK

**Keywords:** Bacterial biofilm, Polymer microarray, High throughput screening, Hemocompatiblility, *Pseudomonas aeruginosa*, *Staphylococcus aureus*

## Abstract

Blood-contacting medical devices play an important role within healthcare and are required to be biocompatible, hemocompatible and resistant to microbial colonization. Here we describe a high throughput screen for copolymers with these specific properties. A series of weakly amphiphilic monomers are combinatorially polymerized with acrylate glycol monomers of varying chain lengths to create a library of 645 multi-functional candidate materials containing multiple chemical moieties that impart anti-biofilm, hemo- and immuno-compatible properties. These materials are screened in over 15,000 individual biological assays, targeting two bacterial species, one Gram negative (*Pseudomonas aeruginosa*) and one Gram positive (*Staphylococcus aureus*) commonly associated with central venous catheter infections, using 5 different measures of hemocompatibility and 6 measures of immunocompatibililty. Selected copolymers reduce platelet activation, platelet loss and leukocyte activation compared with the standard comparator PTFE as well as reducing bacterial biofilm formation *in vitro* by more than 82% compared with silicone. Poly(isobornyl acrylate-co-triethylene glycol methacrylate) (75:25) is identified as the optimal material across all these measures reducing *P. aeruginosa* biofilm formation by up to 86% *in vivo* in a murine foreign body infection model compared with uncoated silicone.

## 1 Introduction

Blood-contacting medical devices, such as vascular catheters and venous access ports, are routinely used in healthcare settings [1,2]. Such devices should be biocompatible, hemocompatible, and resistant to surface-initiated blood coagulation processes and adverse immune reactions [3]. However, devices such as central venous catheters (CVCs) are associated with unacceptably high levels of treatment complications including occlusion, thrombosis and infection. Typically, catheter-related bloodstream infections (CRBSIs) are the most frequent, lethal, and costly complications observed. In the US alone, an estimated quarter of a million CVC-associated infections are responsible for over 30,000 preventable deaths per year [[4], [5], [6]].

Once inserted into the vasculature, biomaterial surfaces become rapidly coated with a blood conditioning film triggering a complex series of closely interlinked events which lead to protein adsorption, platelet and leukocyte adhesion/activation, complement activation, coagulation and thrombosis [7,8]. Activation of the complement and blood clotting cascades drive inflammatory and thrombotic reactions including the generation of anaphylatoxins, such as complement C3a, C5a and bradykinin, thrombin from prothrombin, and the activation of leukocytes and platelets [9], which can collectively cause serious harm to the patient via heart attack or stroke [10,11].

Blood conditioning also impacts on infecting micro-organisms colonising the device either immediately following implantation or *via* hematogenous spread during bacteremia. Common bacterial pathogens causing CVC-associated infections include Gram positives such as *Staphylococcus aureus* and *Staphylococcus epidermidis* and Gram negatives including *Pseudomonas aeruginosa* and *Klebsiella pneumoniae* [12]. Although many different microbial pathogens are capable of forming biofilms on unconditioned CVC surfaces, their coating with blood proteins such as fibrinogen provides additional options for adherence *via* specific bacterial cell surface receptors that facilitate attachment and subsequent biofilm formation [13,14]. For example, pathogens such as *Staphylococcus aureus* can generate an extracellular matrix from the coagulase-dependent conversion of fibrinogen to fibrin [15]. Biofilm formation promotes bacterial survival by shielding infecting pathogens from host immune defences and reducing their susceptibility to antimicrobial agents. Furthermore, the emergence of multi-antibiotic resistant pathogens constitutes a global threat to patient well-being [16]. Thus, generating resistance to bacterial colonization and biofilm development within complex, blood contact environments continues to pose a challenging problem for the biomedical device field. New approaches that reduce the incidence of infection and aid promotion of antibiotic stewardship are required in order to improve patient outcomes.

Loading medical devices with antimicrobial agents has had limited success in mitigating infections, largely due to depletion during use, passivation due to biomolecular adsorption or toxicity [[17], [18], [19], [20], [21]]. Materials that have a molecular structure that inherently resists bacterial biofilm formation by preventing bacterial attachment and/or subsequent biofilm development offer an alternative approach. To date, a number of different surface chemistries have been explored including low-fouling surfaces such as poly(ethylene glycol) (PEG) [22], zwitterionic based materials [[23], [24], [25], [26]] and weakly amphiphilic polyacrylates employing hydrocarbon side chains [[27], [28], [29]]. However, despite some promising *in vitro* and *in vivo* testing successes in animal infection models, clinical efficacy has yet to be demonstrated with respect to infection prevention in blood contacting environments. Thus, there remains a need for the discovery of novel materials that are both resistant to biofilm formation and hemocompatible.

**Fig. 1:**
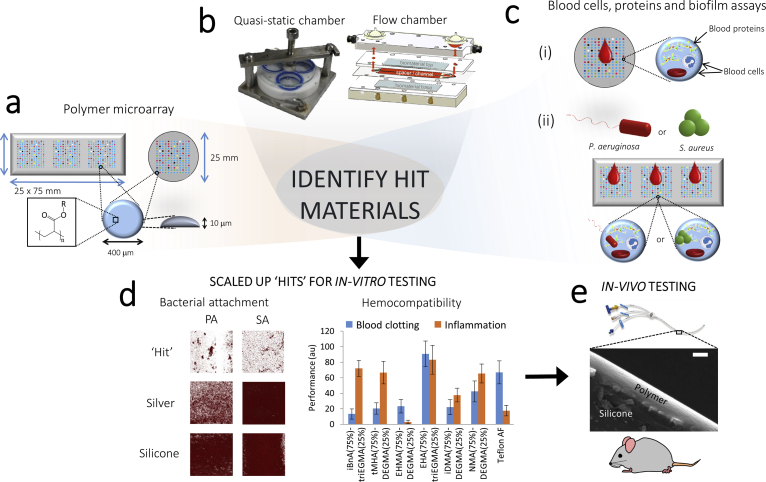
An overview of polymer microarray screening and scale up of ‘hit’ material compositions for *in vitro* and *in vivo* testing. (**a**) Polymer microarray printing using a robotic contact printer on pHEMA coated round and rectangular slides. (**b**) Quasi-static and flow chambers used for human whole blood incubation. (**c**) (i) Quantification of platelet, leukocyte, fibrinogen, IgG, complement C3a binding/adsorption to each microarray polymer spot after 2 h incubation with whole blood; (ii) Incubation of microarrays conditioned in blood for 2 h with *P. aeruginosa* or *S. aureus*. (**d**) Confocal microscope images showing surface bacterial coverage of the lead ‘hit’ material coated onto silicone catheter compared to commercially available silver and uncoated silicone catheters; evaluation of haemostasis and inflammation markers on scaled up hit materials. (**e**) Coating of scaled up ‘hit’ compositions on silicone catheters (confirmed by SEM) and *in-vivo* testing of lead material in a murine foreign body infection model.

High throughput screening approaches have been applied to discover polymeric materials with desirable biological properties including for stem cell culture [[30], [31], [32]], antimicrobial properties [33] and prevention of bacterial biofilm formation [28]. Here we describe a screen for hemo- and immune-compatible copolymers capable of resisting bacterial biofilm formation by employing a multifaceted high throughput microarray screening methodology (Fig. 1) [34,35]. Selected hits were scaled up for further *in vitro* and *in vivo* analysis for selection of an optimal hemo-compatible, anti-biofilm material.

## 2 Results and discussion

### 2.1 Array preparation

We selected a series of weakly amphiphilic monomers from a library of 16 acrylates and methacrylates (Figure SI1, 1-16) that had previously been shown to resist biofilm formation when polymerized [[27], [28], [29],^36^], and have also been shown to permit the permeation of antimicrobials for the creation of dual-functional anti-biofilm and anti-microbial coatings [37]. Since PEG based materials have been widely studied for their ability to prevent fouling by both blood proteins and host cells [22], monomers 1–16 were each combinatorially mixed with acrylate glycol monomers of varying chain lengths (Figure SI1, A-D) at volume ratios of 100:0 (homopolymers), 75:25, 50:50, 25:75 and 0:100 and copolymerized. Zwitterionic polymers have also been proposed as bacterial/protein resistant and hemocompatible materials, thus suitable monomers were included on the array for comparison (Figure SI1, E-G) [24,25]. Together, PEG and zwitterionic materials are representative of the broad class of highly hydrophilic anti-fouling materials. In contrast, the weakly amphiphilic monomers are relatively hydrophobic (water contact angles 70–80°), but also resist bacterial biofilm formation via an anti-attachment mechanism that does not rely upon bacterial killing [28]. The combined monomer solutions were printed in triplicate on a single poly(hydroxy ethyl methacrylate) (pHEMA) coated glass slide for biofilm assays and a single array was printed onto a pHEMA coated coverslip for the hemocompatibility assay (Fig. 1a). After printing the monomers were irradiated with UV light to initiate polymerisation *via* a photo-initiator that had been included in the formulation to generate a polymer microarray with 645 unique materials.

### 2.2 Hemocompatibility and biofilm screen

To obtain an *in vitro* assessment of hemocompatibility for all 645 polymers, the polymer microarray was incubated with whole human blood for 2 h in quasi-static conditions, with no external flow applied to the sample but where the sealed vessels were placed on a rotating platform to avoid blood sedimentation (Fig. 1b). Each polymer spot was then analysed to determine platelet adhesion, fibrinogen, IgG and complement adsorption and leukocyte attachment as a preliminary measure of potential hemo- and immune-compatibility (Figure SI2).

The total amount of the adsorbed protein layer was quantified on each polymer by XPS (quantified based on surface nitrogen composition [38]) (Figure SI2a). Copolymers of triEGMA exhibited the lowest protein adsorption whereas the material with the lowest protein adsorption of 0.2 nm was poly (isobornyl acrylate (iBnA) (25%):triEGMA (75%)). In contrast, copolymers of butyl methacrylate (BuMA), hexyl acrylate (HA) and hexyl methacrylate (HMA) exhibited thicker protein layers, with the polymer poly(HA (75%):triEGMA (25%) exhibiting the highest protein adsorption of 2.5 nm.

Platelet adhesion (Figure SI2b), a key step in supporting the blood clotting cascade, was an order of magnitude (10^2^ vs 7.1 × 10^3^ AU) less than the platelet adhesion measured on polytetrafluoroethylene (PTFE; Teflon) for all polymers assessed. PTFE is commonly used as a comparator for platelet adhesion and overall hemocompatibility and is used to make vascular grafts due to its low surface energy [39]. Reduced platelet adhesion was observed on copolymers of methyl ether terminated poly(ethylene glycol) methacrylate monomers (H3C-PEGMA). Higher adhesion was observed on copolymers of decylmethacrylate (DMA) and iBnA. However, the highest platelet adhesion observed on any of polymers was on a poly(norbornyl methacrylate copolymer (i.e. (NMA) (50%): HO-PEGMA (50%)). For all criteria assessed the acrylates with hydrocarbon pendant groups present on the microarray outperformed the low-fouling PEG and zwitterionic materials tested. This suggests that we had identified a polymer sub-library composed of materials with low-fouling or anti-biofilm properties with significant potential for use in blood contacting environments. It is important to note that the performance of PEG and zwitterionic materials is dependent on their conformation and, therefore, the fabrication process [40]. It is likely that the preparation of these materials by *in-situ* polymerisation did not produce the optimal conformation (ie brush structure) to achieve high level anti-fouling performance. Furthermore, a key aspect of this study was the selection of polymeric formulations, the production of which could be readily scaled to quantities required for the industrial manufacture of medical devices. This was previously achieved for the prevention of bacterial biofilm using a simple and scalable polymerisation approach. Coatings produced from this material are performing well clinically as coatings for urinary tract catheters [41,42]. This gives confidence that scale-up of ‘hit’ formulations from the present screen would result in similar biological performance as was measured on the array format.

Fibrinogen adsorption was also assessed as a precursor to biomaterial-induced thrombosis and because it is targeted by *S. aureus* strains that express fibrinogen-binding proteins [15]. Reduced fibrinogen adsorption was observed on numerous polymers compared with the PEGMA and zwitterionic controls in Figure SI2c. Copolymers of DMA, iBnA, trimethylcyclohexyl methacrylate (tMCHMA) and DEGMA all exhibited low fibrinogen adsorption. In the case of DMA and iBnA, the low fibrinogen adsorption contrasted with the higher platelet adhesion seen in Figure SI2b.

As an assessment of the adherence of blood immune system components, leukocyte attachment, IgG adsorption and C3b adsorption to the different polymers was quantified. Reduced adhesion of both leukocytes and IgG was observed on copolymers of DEGMA and triEGMA compared with HO-PEGMA and H_3_C-PEGMA (Figure SI2d-e). In contrast, the lowest C3b adsorption was observed for the homopolymer of HO-PEGMA and its associated copolymers (Figure SI2f). Copolymers of BuMA, iDMA and NMA also exhibited low C3b adsorption whereas the zwitterionic materials exhibited relatively high levels of C3b adsorption in comparison with the poly(ethylene glycol) (PEG)-based materials.

**Fig. 2:**
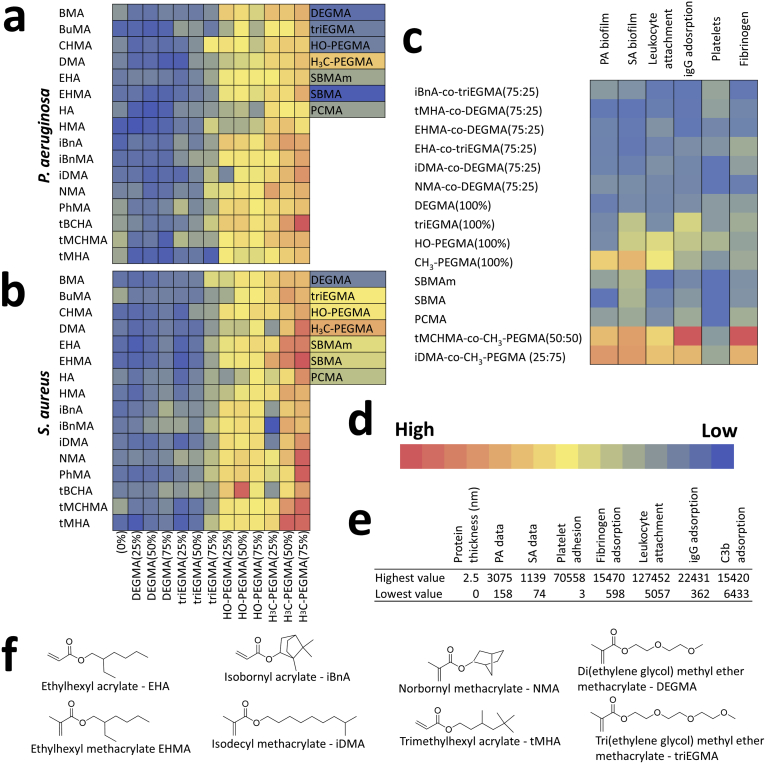
High throughput biological screening of the polymer library and selection of hits. (**a-b**) Intensity map representations of fluorescence readouts (au) of biofilm formation on each polymer spot on the microarray for **a**) *P. aeruginosa* and **b**) *S. aureus*. **c**) Intensity scale of fluorescence values compiling a subset of biological measurements taken for 6 selected ‘hit’ copolymers (first 6 formulations listed), demonstrating the biofilm resistance and hemocompatibility of hit materials across the multiple parameters assessed (biofilm formation (**a** and **b**), leukocyte attachment, IgG adsorption, platelet adhesion and fibrinogen adsorption). Measurements for the entire polymer library are shown in Figure SI2. Values for control non-fouling materials and 2 materials with poor biological performance are also shown. **d**) Intensity scale used for intensity maps. **e**) The numerical high and low values (au) for each biological screen. **f**) The chemical structures of the monomers used to make the ‘hit’ copolymer formulations. The structures of all monomers used in the study are shown in Figure SI1.

The suitability of a polymer for blood contacting medical devices relates not only to bio-, immuno- and hemo-compatibility, but also to its resistance to microbial colonization and subsequent biofilm formation. Consequently, we selected both a Gram negative (*Pseudomonas aeruginosa*) and a Gram positive (*S. aureus*) pathogen known to cause difficult to treat CVC-related bloodstream infections for high throughput screening [17,18]. Both these bacterial species are multi-antibiotic resistant ESKAPE pathogens listed by the World Health Organisation as posing the greatest risk to human health [43]. Increased bacterial biofilm formation was observed for both species on silicone and silver hydrogel coated catheters after blood conditioning (Figure SI3a). Polymer microarrays were incubated for 2 h in whole blood under static conditions prior to incubation with each fluorescently labelled pathogen (Fig. 1c). Intensity maps of the resulting fluorescence measurements are shown in Fig. 2a–b, where the most promising biofilm resistant materials were copolymers with 25% triEGMA or DEGMA. The hits for each bacterial species is shown in Figure SI3b and c.

**Fig. 3:**
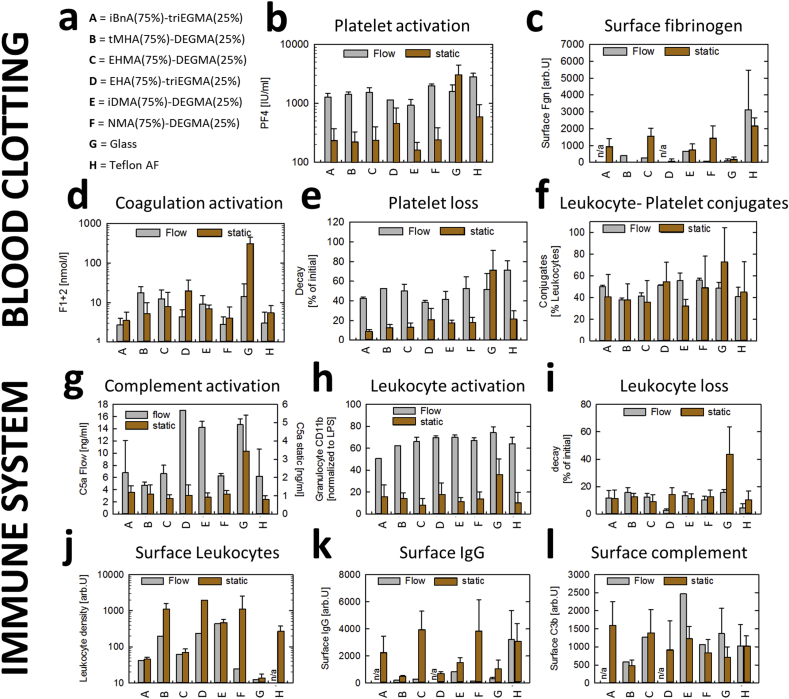
Blood clotting and immune system activation by scaled up selected copolymer formulations under flow () and quasi-static () conditions. **a**) List of materials. All monomer acronyms are listed in Figure SI1. (**b-f**) Blood clotting mediator assays: **b**) platelet activation measured by platelet factor 4 (PF4), **c**) fibrinogen adsorption, **d**) coagulation activation measured by prothrombin fragment 1 + 2 (F1+2), **e**) platelet decay assay, **f**) leukocyte-platelet conjugate assay, (**g-l**) immune component activation assays to assess: **g**) complement activation measured as complement C5a, **h**) leukocyte activation assay using granulocyte CD11b marker normalized to lipopolysaccharide, **i**) leukocyte loss assay, **j**) surface leukocyte density, **k**) IgG surface adsorption, **l**) complement C3b surface adsorption. Error bars equal ±1 standard deviation unit (n = 3). Statistical comparison of measurement shown in Fig. 4 and Figure SI5.

With the exception of the homopolymer of HMA with *P. aeruginosa*, all the DEGMA or triEGMA copolymer formulations outperformed their homopolymer counterparts for both bacterial species. Addition of 25% (v/v) DEGMA or triEGMA to copolymer formulations improved the overall ability of the resulting polymer to resist biofilm formation. However, increasing the DEGMA/triEGMA content to 75% (v/v) generally reduced biofilm resistance (Fig. 2a–b). Furthermore, while the zwitterionic polymer sulfobetaine methacrylate (SBMA) exhibited high resistance to *P. aeruginosa*, the weakly amphiphilic polymers outperformed both the PEG and zwitterionic controls in reducing *S. aureus* biofilm formation. The primary action of these acrylate polymers does not involve killing as very little growth inhibition is observed when the bacteria are cultured in contact with the polymer [28].

*P. aeruginosa* and *S. aureus* biofilm formation on the homopolymers of the ethylene glycol monomers increased with increasing chain length; the lowest biofilm levels were observed on poly(DEGMA). The highest bacterial biofilm coverage for both species was observed on poly(H_3_C-PEGMA), with biofilm levels observed to be significantly (p < 0.024) higher than on poly(HO-PEGMA) for both bacterial species. This suggested that a hydroxyl terminal group on a PEG chain resisted bacterial biofilm formation more effectively than a methyl group for a blood conditioned sample. This may be attributable to differences in hydrophobicity and/or chain conformation that, in turn, modulate the composition of adsorbed blood cell/protein conditioning layer [44]. Although the overall protein layer on poly(HO-PEGMA) was thicker than on poly(H_3_C-PEGMA) (2.11 nm compared with 1.32 nm; Figure SI2b), less fibrinogen was adsorbed. This suggests that the identity of an adsorbed protein on a surface, rather than the amount present is the key to determining how much biofilm forms on a material. This is consistent with the greater level of biofilm formed on H_3_C-PEGMA by *S. aureus* compared with *P. aeruginosa* given that the latter lacks specific fibrinogen receptors.

For 15 of the 16 monomers tested, biofilm formation was reduced by the addition of short chain glycol monomers, with lower bacterial fluorescence values observed for the DEGMA or triEGMA copolymers (except at 75%), when compared with the associated homopolymer (Fig. 2a and b). Thus, it was hypothesised that the mechanism by which the weakly amphiphilic acrylates resist bacterial attachment is different to that of the PEG alternatives. This conclusion was supported by the observation that they exhibited very different affinities for water as indicated by their higher water contact angles [27]. The enhanced resistance to biofilm formation achieved by combining the two monomer types suggests a synergistic combination of two distinct mechanisms, which likely includes modulating the composition of the proteins adsorbed during blood-conditioning.

Each of the eight different result sets from the microarray data were assessed for linear correlations by measuring the coefficient of determination (R^2^) (Figure SI3d). Correlations where R^2^ > 0.3 are shown in Figure SI4. A linear correlation was observed between the attachment of both bacterial species to the materials library (R^2^ = 0.55, Figure SI4a), consistent with previous studies [28]. The highest correlation between two datasets across the entire polymer library was the level of fibrinogen and IgG adsorption (R^2^ = 0.75). An association between fibrinogen adsorption and an immunological response has been previously observed [45], and enhanced adsorption of fibrinogen in the presence of IgG has also been reported [46]. However, previous comparisons of fibrinogen and IgG adsorption from blood plasma onto varied surface coatings did not reveal a strong correlation [47,48]. However, the polyacrylates contained in the polymer microarrays within this study have molecular structures significantly different to the plasma polymer films and poly(vinyl alcohol) membranes studied previously. This suggests that the correlation between fibrinogen and IgG adsorption observed in these studies may be limited to the chemical space represented by the polymer microarrays. The absence of a correlation between platelet attachment or C3b adsorption and any of the other biological parameters studied suggests that these processes occur independently of the biological systems being investigated [49]. Only a weak correlation between leukocyte attachment and IgG adsorption was observed. This contrasted with findings of previous literature studies, which reported that fibrinogen, rather than IgG, promoted leukocyte adhesion [50,51] although differences in the conformation of adsorbed proteins that may influence cellular interactions cannot be ruled out [11].

Bacterial attachment also correlated weakly (R^2^ = 0.3–0.5) with leukocyte attachment, IgG and fibrinogen adsorption but not with protein thickness, C3b adsorption and platelet adhesion (Figure SI3d). These results are consistent with the evidence that specific blood components rather than protein layer thickness are the defining parameters for biofilm formation on these polymers. *S. aureus* for example has multiple cell wall receptors for fibrinogen (e.g. ClfA, ClfB, FnbpA) and IgG [52].

Monomers with branched long chain hydrocarbon moieties, such as iDMA and EHA, featured frequently within the low biofouling material compositions. This suggests that, within the pendant groups of the polymers, the molecular rigidity associated with cyclic structures is less important for preventing bacterial attachment to the blood conditioned polymer surfaces, when compared with previous work conducted in protein free media [29]. However, some cyclic monomers, such as iBnA, also contributed to materials with both low blood component adsorption and resistance to bacterial biofilm formation.

The fluorescence (*F*) values for both bacterial species for each blood exposed polymer were averaged to provide a measure of combined bacterial resistance to biofilm formation. The copolymer with the greatest overall resistance was poly(trimethylhexyl acrylate(75%):DEGMA(25%)) with an average *F* value of 0.016 au. This was similar to the *F* value observed on both p(HO-PEGMA) (*F* = 0.162) and three zwitterionic polymers (i.e. sulfobetaine methacrylamide, sulfobetaine methacrylate and phosphorylcholine methacrylate) *F* = 0.142–0.203 au), suggesting that for this assay, the copolymer more effectively reduced biofilm formation than the common control anti-fouling polymers. Meanwhile, by exhibiting an *F* = 0.841 au, poly(isodecyl methacrylate) (iDMA) (25%):methyl terminated poly(ethylene glycol) methacrylate(H_3_C-PEGMA) (75%)), was the material with the highest overall biofilm formation for both bacterial species.

### 2.3 Hit selection

Polymer formulations were selected for further study by considering the combination of both their bacterial biofilm resistance and blood component adsorption microarray data (Fig. 2 and SI2). The six hit formulations initially selected alongside control samples are listed in Fig. 2c, together with an intensity scale showing the respective values for each material relative to each biological parameter quantified. All hit formulations offered low biological responses for all parameters (i.e. > 83% reduction compared with the highest response measured). However, two copolymers that exhibited the lowest responses to all biological parameters were poly(iDMA(75%): diethylene glycol methyl ether methacrylate (DEGMA) (25%)) and poly(norbornyl methacrylate (75%): tri(ethylene glycol) methacrylate (triEGMA) (25%)). Copolymers containing the short chain glycols (DEGMA and triEGMA) exhibited overall low biological parameters when compared with those containing the PEGMA monomers. Therefore, only DEGMA and triEGMA copolymers were selected for further evaluation. Copolymers were selected in preference to homopolymers, as the homopolymers typically were brittle due to relatively high glass transition temperatures that were lowered after the addition of a co-monomer (associated with the inclusion of glycol monomers), which improved material flexibility [42].

### 2.4 In vitro assessment of scaled-up hit formulations

Since the polymer microarray format is unsuitable for *in vitro* investigations of the activation of blood clotting and inflammatory mediators, the synthesis of promising hemocompatible and biofilm resistant copolymers was scaled-up to approximately 100 g quantities using thermally-induced catalytic chain transfer polymerisation [42]. The resulting materials were characterized by gel permeation chromatography (GPC) and nuclear magnetic resonance (NMR) (Figure SI6-11 and Table SI1). The isolated and purified product copolymers were dip-coated onto class coverslips to form thin films. This sample format allowed the assessment of ‘hit’ materials without the influence of neighbouring spots. Blood clotting and immune system activation assays (Fig. 1d) on the scaled-up copolymers were carried out under both quasi-static (Figure SI12) and flow conditions (Figure SI13) as the shear stress induced by blood flow can cause conformational changes in blood proteins that promote protein adhesion and blood clotting [53]. No visual evidence of delamination was observed and in all cases the coatings were observed to be present after biological assays. Platelet activation and loss (Fig. 3b and e) were the only blood clotting parameters affected by flow where the introduction of shear stress markedly increased the responses observed. Leukocyte-platelet conjugate formation, quantified by flow cytometry via the thrombocyte-specific surface marker CD41a, is a useful measure of surface-mediated coagulation and immune system activation [7,54]. For this assay, the behaviour of the six copolymer formulations matched that of Teflon AF (Fig. 3f).

As an assessment of immune system activation, leukocyte, complement and IgG assays were conducted on the 6 selected copolymers in both flow and static conditions (Fig. 3g–l). As a general trend, all of the surfaces investigated displayed higher complement C5a release and leukocyte activation (CD11b) under flow (Fig. 3g–h). Copolymers poly(ethylhexyl acrylate(EHA) (75%): tri(ethylene glycol) methacrylate (triEGMA) (25%)) and poly(isodecyl acrylate (iDMA) (75%):diethylene glycol methyl ether methacrylate (DEGMA) (25%)) exhibited increased C5a release (2.7 and 2.4 fold higher, respectively) under flow but not under static conditions compared with Teflon AF (Fig. 3g). Each of the copolymers showed minimal leukocyte loss (Fig. 3i) comparable with that of Teflon AF whereas leukocyte density was higher under static conditions compared to flow conditions for the majority of surfaces tested (Fig. 3j). Surface IgG and complement assays produced more variable results with three copolymers showing a comparable response and three showing low surface IgG adsorption compared with Teflon AF (Fig. 3k–l).

Notably, all materials except poly(NMA-co-DEGMA) (75:25) offered a significant (p < 0.05) reduction in platelet activation compared with Teflon, while poly(iBnA-co-triEGMA) (75:25) also produced a significant (p < 0.05) reduction in platelet loss. When comparing with all other polymers, poly(iBnA-co-triEGMA) (75:25) induced signifcantly less leukocyte activation and exhibited lower numbers of surface associated leukocytes whilst poly(EHA-co-triEGMA) (75:25) was responsible for significantly less leukocyte loss, although this polymer caused significantly higher complement activation. Poly(NMA-co-DEGMA) (75:25) exhibited signficantly lower numbers of surface associated leukocytes.

**Fig. 4:**
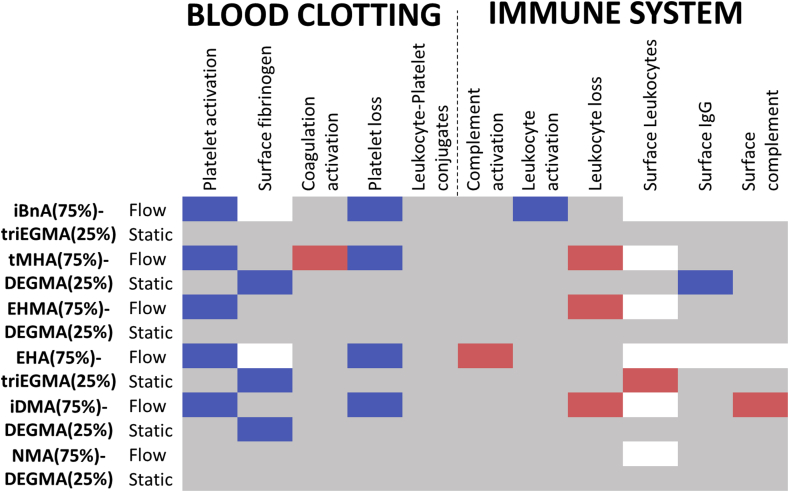
Statistical analysis (student's t-test) of blood clotting cascade and immune system activation measurements on scaled up selected copolymer formulations under flow and quasi-static conditions. Individual datasets shown in Fig. 3. Squares coloured blue or red indicate samples where a significant (p < 0.05) **decrease** or **increase** was observed, respectively, compared to PTFE (comparison with other polymers is shown in Figure SI5). Grey squares indicate no significant difference. Squares coloured white were due to an error in the measurement acquisition for a particular sample. Flow or static conditions are indicated. (For interpretation of the references to colour in this figure legend, the reader is referred to the Web version of this article.)

When the measured parameters were directly compared (Fig. 4), poly(isobornyl acrylate (iBnA)-co-triEGMA) (75:25) achieved overall the lowest thrombogenic and immune/inflammatory responses as the only material showing a statistically significant reduction in platelet activation, platelet loss and leukocyte activation compared with PTFE.

The 6 lead materials were coated onto catheter sections (ca. 50 μm thick, Figure SI14) and assessed in *in vitro* bacterial biofilm assays with *P. aeruginosa* and *S. aureus* after blood pre-conditioning for 2 h as a model of the initial fouling expected on devices after insertion into the body (Fig. 1d). Bacterial biofilm coverage was quantified by confocal microscopy and image analysis (Figure SI15). All of the copolymer formulations exhibited substantially lower bacterial surface coverage than uncoated silicone or commercial silver coated catheter segments, the materials currently used clinically. Poly(iBnA-co-triEGMA) (75:25) showed the lowest level of biofilm formation for both pathogens, 5.5 ± 2.8% and 15.1 ± 4.4%, respectively, corresponding to an overall reduction of 93% and 82% compared with the uncoated silicone catheter (Figure SI15). This result compares favourably with the 97% reduction in bacterial attachment reported in the literature in the absence of blood conditioning using the best material from that study; poly(ethylene glycol dicyclopentenyl ethyl acrylate-co-DEGMA(74:26)) [28]. Thus, the combination of two monomers with different but complementary properties was able to produce a multi-functional material that was both hemocompatible, immunocompatible and resistant to bacterial biofilm formation after exposure and potential fouling by blood components. This material makes use of the anti-fouling properties of ethylene glycol moieties [22], whilst also preserving the anti-biofilm properties of iBnA. In the case of the weakly amphiphilic iBnA monomer, although the mechanism by which this class of monomers prevents bacterial biofilm formation has not been not fully elucidated, it is likely to involve a combination of bacterial sensing of the material surfaces and the physicochemical factors in the near-surface environment, which in this case depend on the action of bulky ester linked hydrophobic pendant groups [29,36]. Live/dead staining of bacterial biofilm grown on the hit polymer compared with silicone showed primarily live cells on both surfaces with a clear reduction in biofilm formation on the former consistent with its biofilm inhibitory properties (Figure SI16). Furthermore, as the coating required no leachable component in order to achieve resistance to bacterial biofilm formation, it is expected to have increased longevity of activity if clinically applied compared with coatings that rely on the delivery of antimicrobials such as silver.

### 2.5 *In vivo* assessment

**Fig. 5:**
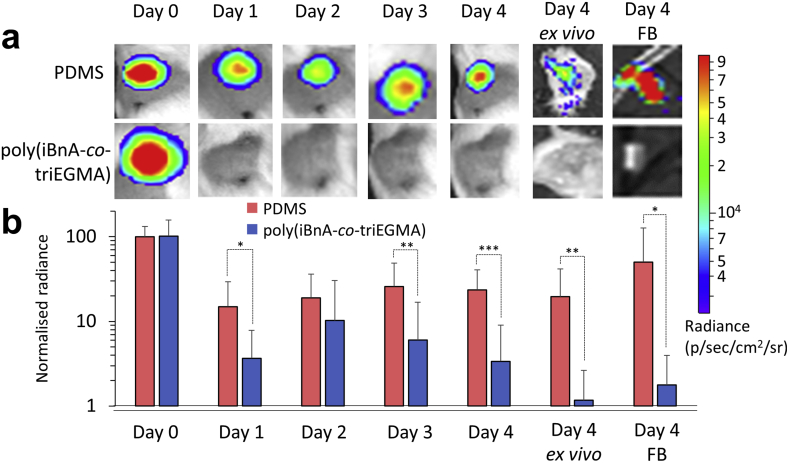
Murine foreign body (FB) infection with *P. aeruginosa* for testing the *in vivo* performance of the ‘hit’ copolymer. a) Luminescent images of the implantation site in live mice over 4 days for uncoated and iBnA-co-triEGMA (75:25) polymer coated silicone catheter segments inoculated with bioluminescent *P. aeruginosa*. The FBs were implanted subcutaneously. Light output from bacteria colonizing the implanted co-polymer coated segments in whole live mice was measured on days 0–4. After the mice were euthanized, the catheter segments were removed and both the surrounding tissues (day 4, *ex vivo*) and the implants (day 4, FB) imaged *ex vivo*. Inset: intensity scale (radiance) where red and blue refer to high and low light outputs respectively. Image dimensions = 16 × 16 mm. b) Quantification of light output (normalized radiance) from uncoated silicone (red) and poly(iBnA-co-triEGMA) polymer coated catheter segments (blue) for *P. aeruginosa*. Error bars show one standard deviation unit, N = 8. Significant differences (Student's unpaired *t*-test) are indicated as * = p < 0.1, ** = p < 0.05 and ***p < 0.01. (For interpretation of the references to colour in this figure legend, the reader is referred to the Web version of this article.)

**Fig. 6:**
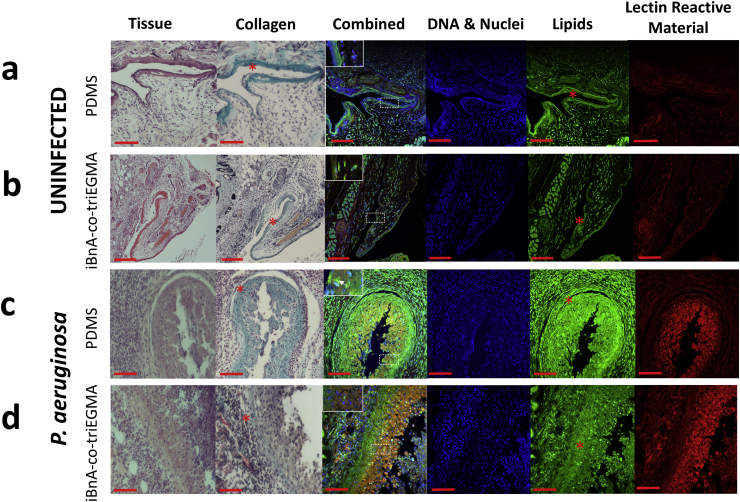
Histological analysis of tissue sections surrounding subcutaneously implanted silicone (a, c) and poly (iBnA-co-triEGMA) catheter segments (b, d) recovered from a-b) control (uninfected) mice and c-d) mice infected with *P. aeruginosa*. Tissue sections were stained from left to right with hematoxylin and eosin (general tissue morphology), Masson's trichome (collagen*), combined (all 5 stains), DNA (DAPI), lipids (FM1-163) and lectins (wheat germ lectin-Alexa 680 conjugate). Of particular note is the high level of lectin reactive staining (red) in the 2 lower righthand panels for c-d indicative of a strong cellular immune response due to the presence of bacteria compared with the sterile upper two right hand control panels (a–b). The insets in the ‘combined’ panel images show localized bacterial foci (c, white arrow) only in the infected mice with silicone implants. (d). There was reduced infiltration of reactive fibroblasts and immune cells to the sterile sites compared with the infection sites. Scale bar equals 50 μm. (For interpretation of the references to colour in this figure legend, the reader is referred to the Web version of this article.)

Given the favourable *in vitro* biological properties of the iBnA-co-triEGMA polymer, we investigated its performance *in vivo* in a murine foreign body (FB) infection model (Fig. 1e). Poly iBnA-co-triEGMA (75:25) coated catheter segment FBs were inserted subcutaneously and inoculated with either a bioluminescent *P. aeruginosa* or *S. aureus* strain and the progress of infection followed over time *via* quantitative whole live animal imaging. Metabolically active bacteria at the infection site were quantified via their light output each day for 4 days post-inoculation (Fig. 5 and Figure SI17). Afterwards the mice were euthanized, the implants were removed, imaged and then subjected to histological analysis of the surrounding tissues (Fig. 6). In the live animals, *P. aeruginosa* exhibited greater than an order of magnitude reduction in luminescence after one day for the coated samples compared with the uncoated samples that persisted for the duration of the experiment (Fig. 5). In contrast to the silicone implants, metabolically active *P. aeruginosa* bacteria failed to seed into the tissues surrounding the poly iBnA-co-triEGMA (75:25) coated FBs (Fig. 5a day 4 *ex vivo*) or colonize the coated implant itself (Fig. 5 day 4, FB).

To examine the qualitative impact of the host response to the copolymer and silicone implants, the tissues from un-infected and infected sites surrounding the implanted catheter segments were excised and stained histologically for the host cellular response and presence of bacterial cells. Fig. 6 shows evidence of fibroblast-mediated collagen deposition in the tissues surrounding the implant sites for both PDMS and the copolymer in both un-infected and infected samples. This was indicative of a foreign body response and tissue repair. Importantly, the poly iBnA-co-triEGMA (75:25) coated polymer samples in common with PDMS and consistent with the *in vitro* blood contact immune assays did not activate an immune response (Fig. 6a and b). A strong cellular immune response was clearly apparent in the tissues of mice infected with *P. aeruginosa* (Fig. 6c and d) as shown by the high level of lectin-staining of immune cells and fibroblasts recruited to the tissues surrounding the implants. However, in contrast to the PDMS implant, no bacteria were present in the tissue samples surrounding the copolymer (Fig. 6 compare c and d, combined staining) consistent with the bioluminescence data and the anti-biofilm properties of poly iBnA-co-triEGMA (75:25)).

Although promising *in vitro* data for *S. aureus* (Figure SI15) on blood conditioned poly(iBnA-co-triEGMA) was obtained, staphylococcal colonization *in vivo* was similar for both coated and uncoated catheter segments sustaining high luminescence over 4 days (Figure SI17). This is consistent with biofilm formation and resistance to immune clearance despite the activation of a robust host response (Fig. S18). Since foreign body responses are associated with the formation of a dense collagen matrix [3], this may be attributable, in part, to the ability of *S. aureus* to bind host tissue proteins such as collagen deposited during host FB responses (e.g. Fig. 6 and S18, collagen stain) *via* specific bacterial surface receptors [52].

## 3 Conclusions

In summary, an *in vitro* multifaceted polymer microarray screen has been developed to discover materials that are hemocompatible, minimally activate the host immune system and refractory to bacterial biofilm formation. Selected copolymers were demonstrated to maintain reduced blood clotting cascade and immune system activation as well as resistance to biofilm formation *in vitro*. The best performing material *in vitro*, poly(iBnA(75%); triEGMA(25%)), reduced *P. aeruginosa* biofilm formation by up to 86% *in vivo* compared with uncoated silicone after 4 days. The data presented highlight the potential of this material as a blood contacting biomaterial that prevents biofilm-centred infections associated with vascular access devices.

## 4 Experimental section

*Materials:* Monomers were purchased from Sigma Aldrich and solvents from Fisher Scientific UK respectively and used as received without further purification. All the copolymer ratios defined in this manuscript are v/v ratios.

*Polymer Microarrays:* Polymer microarrays were prepared as previously described [28]. Monomers 1–16 were mixed with glycol monomers at ratios of 1:0, 3:1, 1:1, 1:3 and 0:1 (v/v). The combined monomer solutions were printed in triplicate as approximately 400 μm diameter spots with a height of ~10 μm. A total of 20 replicate arrays of 645 materials were typically prepared in 6–10 h.

*Coverslip Dip coating:* Epoxy functionalised glass slides (Arrayit) were dip coated with 6% w/v pHEMA in 19:1 ethanol:water (v/v) 4 times in sequence with a withdrawal speed of 2 mm/s and 10 min drying between dips using a dip coater (HO-TH-01, Holmarc, India). Coatings were kept in ambient conditions at room temperature prior to testing.

*XPS Analysis:* X-ray photoelectron spectroscopy (XPS) was conducted using a Kratos Axis Ultra spectrometer with a mono-chromated Aluminium X-ray gun (AlKα = 1253.6 eV) and a charge-compensating electron flood gun. Photoelectrons were sampled from a 110 μm diameter (aperture) in the centre of each polymer spot in snapshot mode for the C1s, N1s and O1s core levels. The acquisition time was limited to 30 s for N1s and 20 s for C1s and O1s scans, using a pass energy of 160 eV. Data analysis was carried out using CASA XPS software (version 2.3.16 PR 1.6).

*Catalytic chain transfer polymerisation:* Bis[(difluoroboryl) diphenylglyoximato] cobalt(II) (100 mg, DuPont) and 2,2′-azobis(4methoxy-2,4-dimethyl valeronitrile) (240 mg, Sigma) were added to a 500 ml flask containing argon. In a typical experiment to prepare a 75%:25% v/v polymer, 75.2 ml of the major monomer, 20.8 ml of minor monomer and 200 ml of toluene were added to the flask. The subsequent reaction solution was then degassed by bubbling with argon for 30 min. The reaction mixture was then raised to and held at 80 °C for 18 h with stirring. The polymerisation was terminated by cooling the contents to room temperature and exposure to air with rapid stirring. The polymer was precipitated in cold methanol and isolated by filtration or decanting the solvent, depending on the physical form of the resultant polymer, before drying under vacuum.

*NMR Analysis: -* ^1^H NMR spectroscopic analyses were recorded at 25 °C using Bruker DPX-300 and AV-400 MHz spectrometers in toluene-d8 or deuterated chloroform (CDCl_3_) (1 mg ml-1) to which chemical shifts were referenced (residual toluene at 2.09 ppm and chloroform at 7.26 ppm). Analysis of the spectra was carried out using ACDLABS 12 software.

*GPC Analysis*: GPC was carried out at a solvent flow at 1 ml min^−1^. A 7 mg ml^−1^ polymer solution in tetrahydrofuran was injected to the GPC system by an autosampler through a series of three columns: one PLgel 5 μm Guard column (Polymer Laboratories) and then two PLgel 5 μm MIXED-C columns (Polymer Laboratories), which were kept at 35 °C. After separation, the polymer fractions were analysed by an IR detector.

*SEM Analysis:* Coating thickness was assessed by scanning electron microscopy (SEM). Coated samples were plunged into liquid nitrogen and fractured using a scalpel blade. Samples were sputter coated with gold for 4 min using a Leica EM SCD005 support coater and imaged using a JEOL 6060LV variable pressure SEM.

*Catheter Section Dip Coating:* Rusch Brilliant Paediatric silicone catheters (Teleflex Medical) size 8 F R, 3 ml and 31 cm length were cut into 1 cm lengths. Samples were activated by O_2_ plasma at 50 W power and 300 mTorr working pressure for 10 min in a custom built reactor [55]. Catheter pieces were dip coated with 30% w/v polymer solution (in DCM) using a dip coater (HO-TH-01, Holmarc, India) before drying under vacuum (<50 mbar) for seven days.

*Bacterial Strains and Growth Media*: The bacterial pathogens used were *P. aeruginosa* PAO1-L and *Staphylococcus aureus* SH1000. These were labelled with either fluorescent proteins or rendered bioluminescent by the introduction of bacterial luciferase (*lux*) genes. The mCherry (pMMR; tetracycline resistant) [56] and mKat (pBS10 -mKat plasmid (erythromycin resistant)) [57] expression vectors were transformed into *P. aeruginosa* and *S. aureus,* respectively, by electroporation and maintained on LB agar plates containing tetracycline (125 μg/ml) or erythromycin (30 μg/ml).

*Biofilm assays:* For assays of biofilm formation on microarray slides and catheter segments, bacteria were grown overnight in RPMI-1640 medium at 37 °C with shaking at 200 rpm after which the cells were harvested by centrifugation. Bacteria were resuspended to an optical density at 600 nm (OD_600_) of 0.01 in 15 ml fresh RPMI-1640 containing either a microarray slides or uncoated silicone or coated catheter segments previously incubated for 2 h with human blood as described. The cultures were incubated at 37 °C with shaking at 60 rpm for 72 h. Slides and catheter segments were washed once with PBS and once with deionized water before imaging. Microarrays were imaged using a Genepix 4200AL fluorescence scanner (Molecular Devices UK Ltd.) using an excitation wavelength of 594 nm and emission filter set at 607–694 nm. Image processing was conducted using Genepix Pro 6.1 software (Molecular Devices UK Ltd.). Catheter segments were imaged by confocal fluorescence microscopy (Zeiss LSM 710) and biofilm surface coverage calculated using the ImageJ plugin, Comstat 2.1 [58]. Live/dead staining was carried out using a LIVE/DEAD™ BacLight™ Bacterial Viability Kit (Molecular Probes) as per the manufacturer's instructions.

*Whole blood incubation assay:* The phlebotomy work was carried out in the Clinical Research Facility, School of Medicine based at Queens Medical Centre, Nottingham University Hospitals as per ethical approval (Ref: BT09052014 SoP SoLS, Faculty of Medicine and Health Sciences Research Ethics Committee). When using the static cell, microarrays were assembled as top and bottom of autoclaved quasi-static incubation chambers made of PTFE, with printed side facing inwards (Figure SI12). Each incubation chamber held 1.95 ml blood with a surface to volume ratio of 3.2 cm^−1^ [59]. The incubation chambers were filled with 0.9% NaCl solution and allowed to equilibrate at 37 °C prior to whole blood exposure. For the flow cell, samples were assembled into the flow cell as depicted in Figure SI13. Blood was incubated at a shear rate of 300 s^−1^ using a spacer of 150 μm.

Venous blood was obtained using a 19G cannula from two healthy volunteers with no known medical conditions and who had not taken any medication 10 days prior to blood donation, with 3 repeats taken for each volunteer. Blood clotting was prevented by addition of 2 (static) or 4 (dynamic) IU/ml heparin and immediately used to fill incubation chambers (pre-warmed to 37 °C), avoiding air bubbles in the chambers. Samples were incubated for 2 h at 37 °C. Following incubation, blood was drained and the slides washed twice with PBS and once with deionized water.

For leukocyte and platelet detection experiments, samples were incubated in 2% paraformaldehyde in phosphate buffered saline (PBS) for 1 h followed by a PBS wash. Leukocyte detection was carried out using a DNA-staining dye Sytox Green (1 μM) with 0.05% Saponin (30–60 min). Surface attached platelets were detected using mouse anti-human CD41a FITC (FACS-antibody, Becton Dickinson) 1:10 in blocking buffer (2% dry milk powder, 0.05% Tween-20 in PBS).

For fibrinogen, IgG and complement C3b detection, samples were incubated in blocking buffer for 30 min. Rabbit anti-human fibrinogen (FITC) (Cedarlane) 1:30 in blocking buffer was used to detect fibrinogen by incubating for 1 h. Goat anti-human IgG (Rhodamine Red X) (Jackson Immuno) 1:50 in blocking buffer was used to detect IgG. Rabbit (rb) anti-human C3b 1:500 (primary antibody) and anti-rb IgG (Alexa 488) 1:200 (secondary antibody) were used to detect C3b.

Complement fragment C5a, prothrombin fragment F1+2 and platelet factor 4 (PF4) were assessed by ELISA (C5a micro, DRG Instruments, Marburg, Germany; Enzygnost F1+2 micro, Siemens, Eschborn, Germany; Zymutest PF4, CoaChrom, Vienna, Austria, respectively) according to the manufacturers’ instructions. Blood cell counts were determined with a cell counter ACT diff (Beckman coulter, Krefeld, Germany). CD11b expression on granulocytes and monocytes and platelet leukocyte conjugate formation were assessed by flow cytometry in a lyse-no-wash protocol (anti CD11b clone ICRF44, Biozol, Eching, Germany, and anti CD41a, (clone HIP8, Becton Dickinson, Heidelberg, Germany; cytometer FACSCalibur, Becton-Dickinson, Heidelberg, Germany).

Fluorescence measurements were performed using a Fujifilm FLA-5100 fluorescence scanner.

*In vivo mouse infection experiments:* All animal work was approved following ethical review at the University of Nottingham and performed under U.K Home Office licence 30/3238. The murine foreign body model was conducted as described previously [28] where mice were implanted subcutaneously with either silicone or poly(iBnA-co-triEGMA) coated catheter segments. Animals were allowed to recover for 4 days prior to inoculation with 1 × 10^5^ constitutively bioluminescent *S. aureus* (strain SH1000) or *P. aeruginosa* (strain PAO1-L CTX:*tac-lux*) respectively into the lumen of longitudinally bisected catheter segments (2.6 mm × 4.2 mm x 7.5 mm). Injection of the bacteria into the lumen ensured that the inoculum was contained proximal to the catheter surface aiding uniformity of infection levels between each mouse and experiment. Mice were imaged under anesthesia using an IVIS spectrum camera (Caliper) after the initial bacterial inoculation to provide a readout of infection level for normalization, and then at 24 h intervals. Infection was tracked over 4 days by assessment of light output for the localization of metabolically active bacteria at the infection site. At day 4 mice were humanely killed and the catheter segment and surrounding tissue harvested. The catheter was then excised from the tissue and the localization of metabolic bacteria determined for both the tissue and the catheter.

*Histological assessment of infection site tissues:* Tissues excised from the infection sites were fixed by incubation in 10% formal saline for 24 h, and then processed for paraffin embedding. 8 μm sections were stained for the evaluation of tissue morphology using hematoxylin and eosin staining. Collagenesis was assessed using Masson's trichrome (light green; Atom-scientific RRSK21-500). The localization of glycoproteins induced via the host innate immune response to infection was determined using a wheat germ agglutinin (WGA)-Alexa 680 conjugate (Thermofisher), incubated 5 μg/ml for 1 h at 37 °C, washed twice PBS and then stained for DNA and lipids using DAPI (5 μg/ml) and FM-143 (5 μg/ml) for 10 min and mounted with Fluoromount (Sigma-Aldrich). Images were taken on a Zeiss 700 confocal microscope.

## CRediT authorship contribution statement

**Taranjit Singh:** Investigation, Formal analysis, Writing - original draft. **Andrew L. Hook:** Methodology, Formal analysis, Data curation, Writing - original draft, Visualization. **Jeni Luckett:** Investigation, Formal analysis, Methodology, Writing - original draft, Visualization. **Manfred F. Maitz:** Methodology, Validation, Writing - original draft. **Claudia Sperling:** Methodology, Validation, Writing - original draft. **Carsten Werner:** Conceptualization, Methodology, Resources, Writing - original draft. **Martyn C. Davies:** Conceptualization, Resources, Writing - original draft, Supervision, Funding acquisition. **Derek J. Irvine:** Methodology, Supervision, Validation, Writing - original draft. **Paul Williams:** Conceptualization, Resources, Writing - original draft, Supervision, Funding acquisition. **Morgan R. Alexander:** Conceptualization, Resources, Writing - original draft, Supervision, Funding acquisition.

## Declaration of competing interest

The authors declare that they have no known competing financial interests or personal relationships that could have appeared to influence the work reported in this paper.
