# Supplementary material for: Discovery of hemocompatible bacterial biofilm-resistant copolymers
Source: Biomaterials. 2020 Nov;260:120312. doi: 10.1016/j.biomaterials.2020.120312 (PMC7534038; doi:10.1016/j.biomaterials.2020.120312)
Supplement: Multimedia component 1 [file mmc1.docx]

Discovery of Hemocompatible Bacterial Biofilm-Resistant Copolymers

**Supplementary information**

**Taranjit Singh^1,5¶^_,_ Andrew L. Hook^1¶^, Jeni Luckett^2^, Manfred F. Maitz^3^, Claudia Sperling^3^**, **Carsten Werner^3^, Martyn C. Davies^1^, Derek J. Irvine^4^, Paul Williams^5§^, & Morgan R. Alexander^1§^***

*^1^School of Pharmacy, University of Nottingham, Nottingham, NG7 2RD, UK ^2^Biodiscovery Institute and School of Medicine, University of Nottingham, UK NG7 2UH ; ^3^Leibniz Institute of Polymer Research Dresden, Max Bergmann Centre for Biomaterials Dresden Hohe Str. 6, D-01069 Dresden; ^4^Department of Chemical and Environmental Engineering, Faculty of Engineering, University of Nottingham, Nottingham, NG7 2RD UK; University of Nottingham, Nottingham, NG7 2RD, UK; ^5^Centre for Biomolecular Sciences and School of Life Sciences, University of Nottingham, Nottingham, NG7 2RD, UK.*

**^¶^** joint first authors

^§^ joint senior authors

*corresponding author


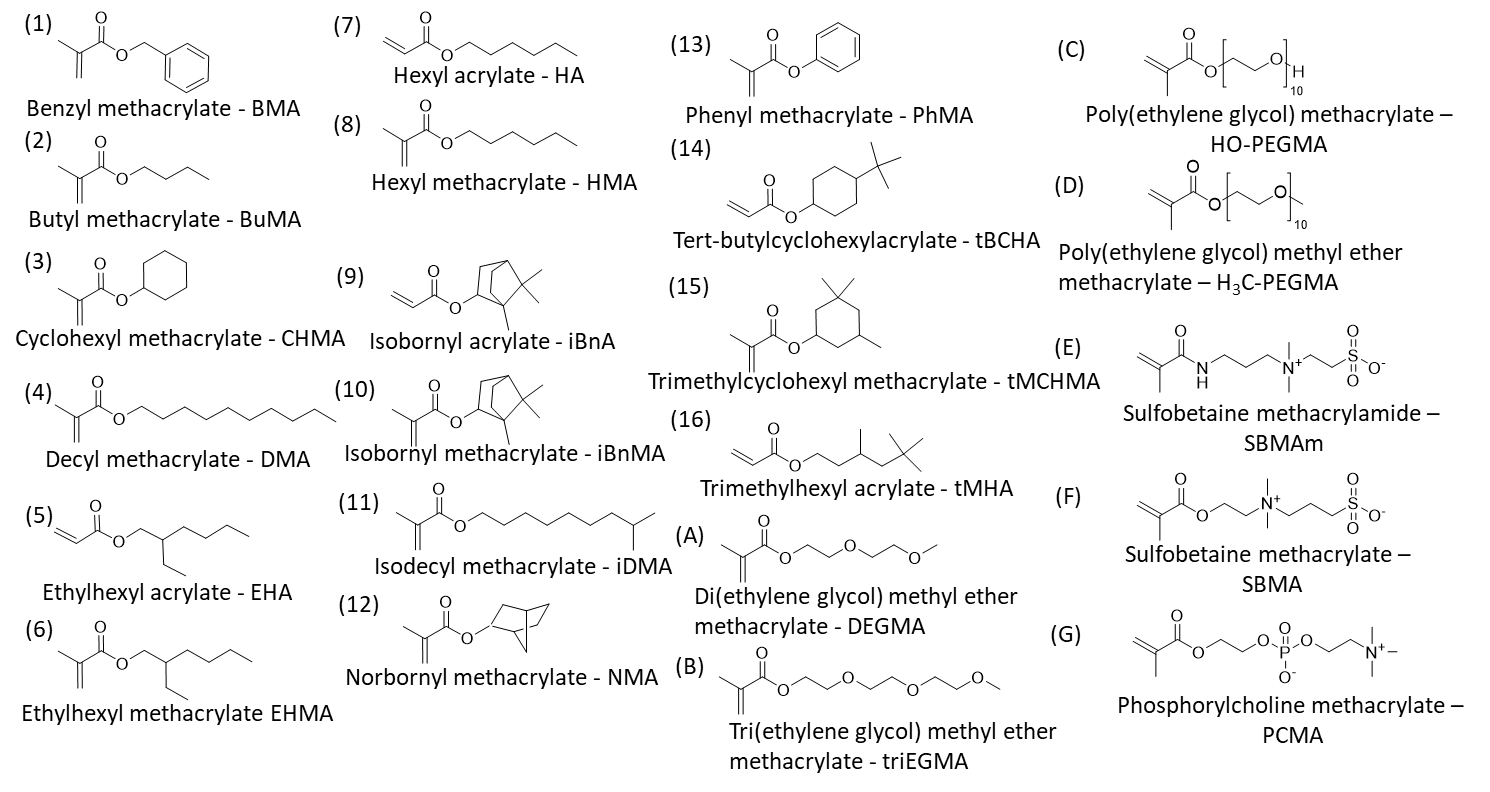


**Figure SI1.** Chemical structures of the monomers used in this study, including their names and abbreviations. Monomers 1-16 consist of monomers previously demonstrated to prevent bacterial biofilm formation, monomers A-D consist of co-monomers with anti-fouling properties associated with the ethylene glycol moiety.

**
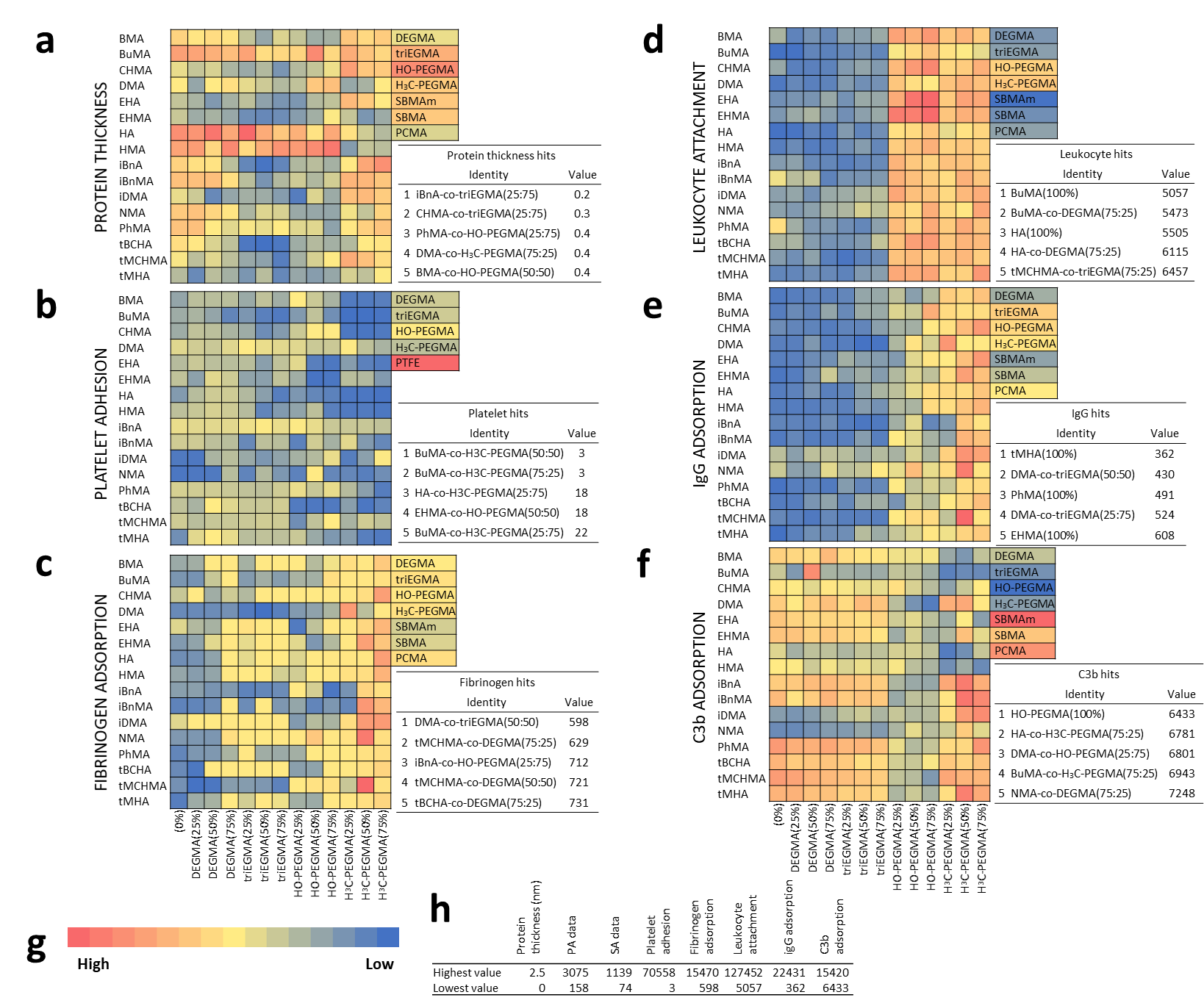
**

**Figure SI2.** Intensity map representations of biological screens on the polymer microarray for a) protein thickness (nm) as measured by XPS and fluorescence measurements of b) platelet adhesion (arbitrary units - au) c) fibrinogen adsorption (au), d) leukocyte attachment (au), e) IgG adsorption (au), and f) C3b adsorption. A table showing the top 5 hits for each category are also shown with the corresponding measured values. g) Intensity scale used for intensity maps. h) The highest and lowest fluorescence values for all fluorescence screens. All monomer acronyms are listed in **Figure SI1**.

**
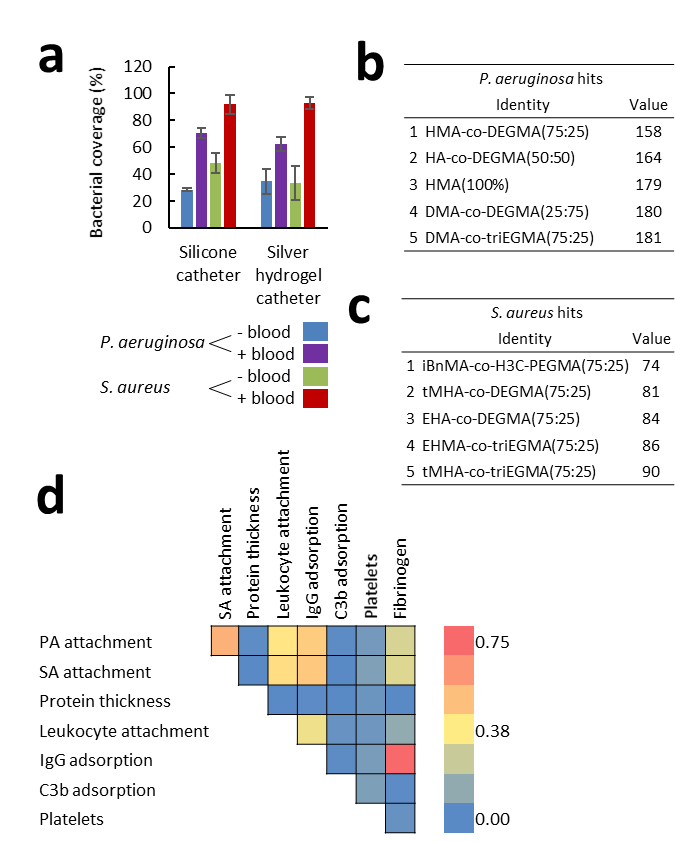
**

**Figure SI3.** Biofilm formation on catheters conditioned with blood for 2h. a) Bacterial surface coverage measured on silicone or silver hydrogel coated catheter segments for *P. aeruginosa* or *S. aureus* with and without blood conditioning. Results provided are the mean of 3 biological replicates, error bars are ± one standard deviation unit. Differences in bacterial coverage with and without blood conditioning are statistically significant (p<0.01) for both bacterial species and both sample types. (b-c) Tables showing the top 5 hits for b) *P. aeruginosa* and c) *S. aureus* with the corresponding measured values. d) Heat map showing the R^2^ values comparing linear correlations between the hemo- immuno- and biofilm parameters assessed for the whole polymer microarray. An intensity scale is provided on the right. Plots used to assess the correlations are shown in **Figure SI4**. All monomer acronyms are listed in **Figure SI1**.

**abc**

**def**

**ghi**

**Figure SI4.** Comparison of microarray readouts where a correlation (R^2^>0.3) was observed. (a-d) The fluorescence due to *P. aeruginosa* (*PA*) versus (a) *S. aureus* (*SA*) fluorescence, (b) leukocyte fluorescence, (c) IgG fluorescence and (d) fibrinogen fluorescence for each polymer within the copolymer library. (e-g) The fluorescence due to *SA* versus (e) leukocyte fluorescence, (f) IgG fluorescence and (g) fibrinogen fluorescence for each polymer within the copolymer library. (h) Comparison of the IgG and Leukocyte fluorescence observed on each polymer within the copolymer library. (i) Comparison of the IgG and fibrinogen fluorescence observed on each polymer within the copolymer library. Values reported are the mean of 3 measurements.

|  |  | Platelet activation | Surface fibrinogen | Coagulation activation | Platelet loss | Leukocyte-Platelet conjugates | Complement activation | Leukocyte activation | Leukocyte loss | Surface Leukocytes | Surface IgG | Surface complement |
| --- | --- | --- | --- | --- | --- | --- | --- | --- | --- | --- | --- | --- |
| **A** | Flow |  |  |  |  |  |  |  |  |  |  |  |
|  | Static |  |  |  |  |  |  |  |  |  |  |  |
| **B** | Flow |  |  |  |  |  |  |  |  |  |  |  |
|  | Static |  |  |  |  |  |  |  |  |  |  |  |
| **C** | Flow |  |  |  |  |  |  |  |  |  |  |  |
|  | Static |  |  |  |  |  |  |  |  |  |  |  |
| **D** | Flow |  |  |  |  |  |  |  |  |  |  |  |
|  | Static |  |  |  |  |  |  |  |  |  |  |  |
| **E** | Flow |  |  |  |  |  |  |  |  |  |  |  |
|  | Static |  |  |  |  |  |  |  |  |  |  |  |
| **F** | Flow |  |  |  |  |  |  |  |  |  |  |  |
|  | Static |  |  |  |  |  |  |  |  |  |  |  |

**Figure SI5**. Results from student’s t-test analysis of blood clotting cascade and immunological activation measurements (**Figure 3**) on scaled up hit polymers. Squares coloured blue or red indicate samples where a significant (p<0.05) **decrease** or **increase** was observed, respectively, compared to all other polymer samples. Grey squares indicate no significant difference. Squares coloured white were due to an error in the measurement acquisition for a particular sample. Flow or static conditions are indicated.

**Table SI1**. GPC and NMR analysis of scaled up polymers. All monomer acronyms are listed in **Figure SI1**. The associated NMR spectra are shown in **Figure SI6-11**.

| Material composition  (Vol/Vol) | Measured composition^a^ | Conversion^a^ | M_w_^b^  (g mol^-1)^ | M_n_^b^  (g mol^-1)^ | Polydispersity^b^ index (PDI) |
| --- | --- | --- | --- | --- | --- |
| iBnA(75%):triEGMA(25%) | 3.5:1 (78%:22%) | 53% | 21,934 | 5,834 | 3.8 |
| tMHA(75%):DEGMA(25%) | 2.5:1 (71%:29%) | 83% | 29,507 | 11,003 | 2.7 |
| EHMA(75%):DEGMA(25%) | 2.9:1 (74%:26%) | 79% | 13,438 | 8,221 | 1.6 |
| EHA(75%):triEGMA(25%) | 2.9:1 (74%:26%) | 68% | 20,074 | 8,872 | 2.3 |
| iDMA(75%):DEGMA(25%) | 2.7:1 (73%:27%) | 55% | 14,378 | 8,253 | 1.7 |
| NMA(75%):triEGMA(25%) | 3.4:1 (77%:23%) | 93% | 15,762 | 8,191 | 1.9 |

**^a^** Determined by NMR analysis, **^b^** Determined by GPC analysis

**Figure SI6**. ^1^H NMR of poly(iBnA-co-triEGMA) (75:25) (400 MHz, toluene-d8) δ ppm 0.53 - 1.01 (m, 36 H) 1.24 - 1.67 (m, 21 H) 3.12 (d, *J*=6.06 Hz, 3 H) 3.23 - 3.33 (m, 2 H) 3.35 - 3.51 (m, 8 H) 3.75 - 4.09 (m, 2 H) 4.18 - 4.48 (m, 2 H) 4.52 (dd, *J*=7.58, 4.04 Hz, 1 H). The peak at 2.09 is due to toluene and was used as a reference. The ratio of hydrogens with neighbouring electron-withdrawing groups (3-4.5 ppm) to hydrogens without electron-withdrawing neighbouring groups (0.5-1.7 ppm) was used to determine composition, noting iBnA contains 1 hydrogen with a neighbouring oxygen and 19 hydrogens with non-electron withdrawing neighbours, whilst triEGMA contains 15 hydrogens with a neighbouring oxygen and 5 hydrogens with non-electron withdrawing neighbours.

**Figure SI7**. ^1^H NMR of poly(tMHA-co-DEGMA) (75:25) (400 MHz, toluene-d8) δ ppm 0.47 - 1.51 (m, 56 H) 3.08 - 3.17 (m, 3 H) 3.30 (br s, 2 H) 3.33 - 3.51 (m, 4 H) 3.59 - 4.03 (m, 8 H). The peak at 2.09 is due to toluene and was used as a reference. The ratio of hydrogens with neighbouring electron-withdrawing groups (3-4.5 ppm) to hydrogens without electron-withdrawing neighbouring groups (0.5-1.7 ppm) was used to determine composition, noting tMHA contains 2 hydrogen with a neighbouring oxygen and 20 hydrogens with non-electron withdrawing neighbours, whilst DEGMA contains 11 hydrogens with a neighbouring oxygen and 5 hydrogens with non-electron withdrawing neighbours.

**Figure SI8**. ^1^H NMR of poly(EHMA-co-DEGMA) (75:25) (400 MHz, toluene-d8) δ ppm 0.58 - 1.43 (m, 56 H) 1.52 - 1.80 (m, 6 H) 3.07 - 3.17 (m, 3 H) 3.23 - 3.50 (m, 6 H) 3.51 - 3.99 (m, 7 H) 4.00 - 4.08 (m, 1 H). The peak at 2.09 is due to toluene and was used as a reference. The ratio of hydrogens with neighbouring electron-withdrawing groups (3-4.5 ppm) to hydrogens without electron-withdrawing neighbouring groups (0.5-1.7 ppm) was used to determine composition, noting EHMA contains 2 hydrogen with a neighbouring oxygen and 20 hydrogens with non-electron withdrawing neighbours, whilst DEGMA contains 11 hydrogens with a neighbouring oxygen and 5 hydrogens with non-electron withdrawing neighbours.

**Figure SI9**. ^1^H NMR of poly(EHA-co-triEGMA) (75:25) (400 MHz, toluene-d8) δ ppm 0.53 - 1.53 (m, 68 H) 3.12 (s, 3 H) 3.30 (br d, *J*=4.80 Hz, 2 H) 3.40 (br s, 8 H) 3.83 (dd, *J*=5.94, 2.40 Hz, 9 H). The peak at 2.09 is due to toluene and was used as a reference. The ratio of hydrogens with neighbouring electron-withdrawing groups (3-4.5 ppm) to hydrogens without electron-withdrawing neighbouring groups (0.5-1.7 ppm) was used to determine composition, noting EHA contains 2 hydrogen with a neighbouring oxygen and 20 hydrogens with non-electron withdrawing neighbours, whilst triEGMA contains 15 hydrogens with a neighbouring oxygen and 5 hydrogens with non-electron withdrawing neighbours.

**Figure SI10**. ^1^H NMR of poly(iDMA-co-DEGMA) (75:25) (400 MHz, toluene-d8) δ ppm 0.43 - 1.83 (m, 70 H) 3.06 - 3.19 (m, 3 H) 3.23 - 3.51 (m, 6 H) 3.56 - 4.10 (m, 7 H). The peak at 2.09 is due to toluene and was used as a reference. The ratio of hydrogens with neighbouring electron-withdrawing groups (3-4.5 ppm) to hydrogens without electron-withdrawing neighbouring groups (0.5-1.7 ppm) was used to determine composition, noting iDMA contains 2 hydrogen with a neighbouring oxygen and 24 hydrogens with non-electron withdrawing neighbours, whilst DEGMA contains 11 hydrogens with a neighbouring oxygen and 5 hydrogens with non-electron withdrawing neighbours.

**Figure SI11**. ^1^H NMR of poly(NMA-co-triEGMA) (75:25) (400 MHz, toluene-d8) δ ppm 0.57 - 1.02 (m, 19 H) 1.08 - 1.82 (m, 21 H) 3.07 - 3.19 (m, 3 H) 3.22 - 3.52 (m, 6 H) 3.80 - 3.99 (m, 2 H) 4.19 - 4.34 (m, 2 H) 4.35 - 4.44 (m, 1 H). The peak at 2.09 is due to toluene and was used as a reference. The ratio of hydrogens with neighbouring electron-withdrawing groups (3-4.5 ppm) to hydrogens without electron-withdrawing neighbouring groups (0.5-1.7 ppm) was used to determine composition, noting NMA contains 1 hydrogen with a neighbouring oxygen and 15 hydrogens with non-electron withdrawing neighbours, whilst triEGMA contains 15 hydrogens with a neighbouring oxygen and 5 hydrogens with non-electron withdrawing neighbours.


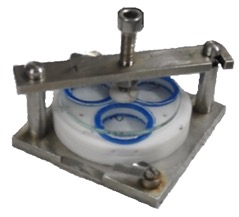


**Figure SI12.** Image of the sample assembly used for measurements conducted under quasi-static conditions.


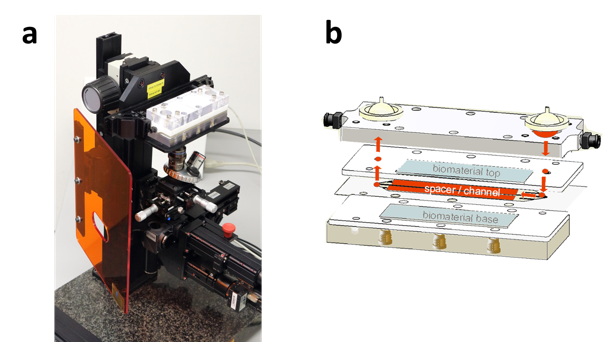


**Figure SI13.** The custom-built flow cell incubation chamber used for blood contact experiments. a) Blood flow chamber mounted on an inverted microscope, b) a detailed schematic representation of various components of the flow chamber, spacer thickness = 150 μm.

**
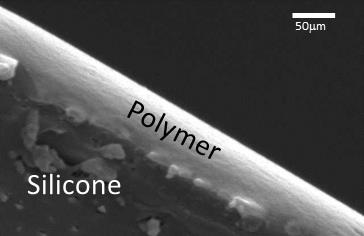
**

**Figure SI14.** Typical SEM image of the cross-section of a coated catheter prepared by fracturing a sample frozen in liquid nitrogen. Scale bar is 50 μm.


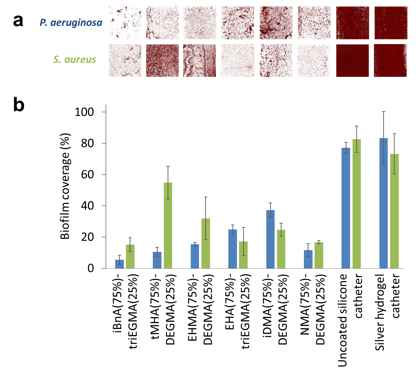


**Figure SI15.** Bacterial surface coverage on copolymer coated silicone catheters conditioned for 2 h in blood following 72 h incubation with *P. aeruginosa* (◼) or *S. aureus* (◼)*.* The monomer composition is displayed. (a) Confocal microscope images (625 x 625 µm) of mCherry labelled *P. aeruginosa* and mKat labelled *S. aureus* biofilms on polymer coated surfaces, uncoated silicone and silver containing catheters. (b) Quantification of biofilm surface coverage (%) for *P. aeruginosa* (blue) and *S aureus* (red). Scales bars equal ± 1 standard deviation unit, N = 3. All monomer acronyms are listed in **Figure SI1**.

**ab**

**cd**

**Figure SI16.** Live/dead staining on (a,b) uncoated silicone catheters and (c,d) hit material poly iBnA-co-triEGMA (75:25) coated onto silicone catheters. The coated and uncoated silicone catheters were incubated in RPMI-1640 media containing *P. aeruginosa* for 72 hours. The surfaces were then stained using BacLight^TM^ bacterial viability kit. Fluorescent images showing fluorescence due to (a,c) SYTO® 9 green-fluorescent nucleic acid stain (stains live bacteria) and (b,d) propidium iodide, which is a red-fluorescent nucleic acid stain (stains dead bacteria). Each image is 625 x 625 μm.


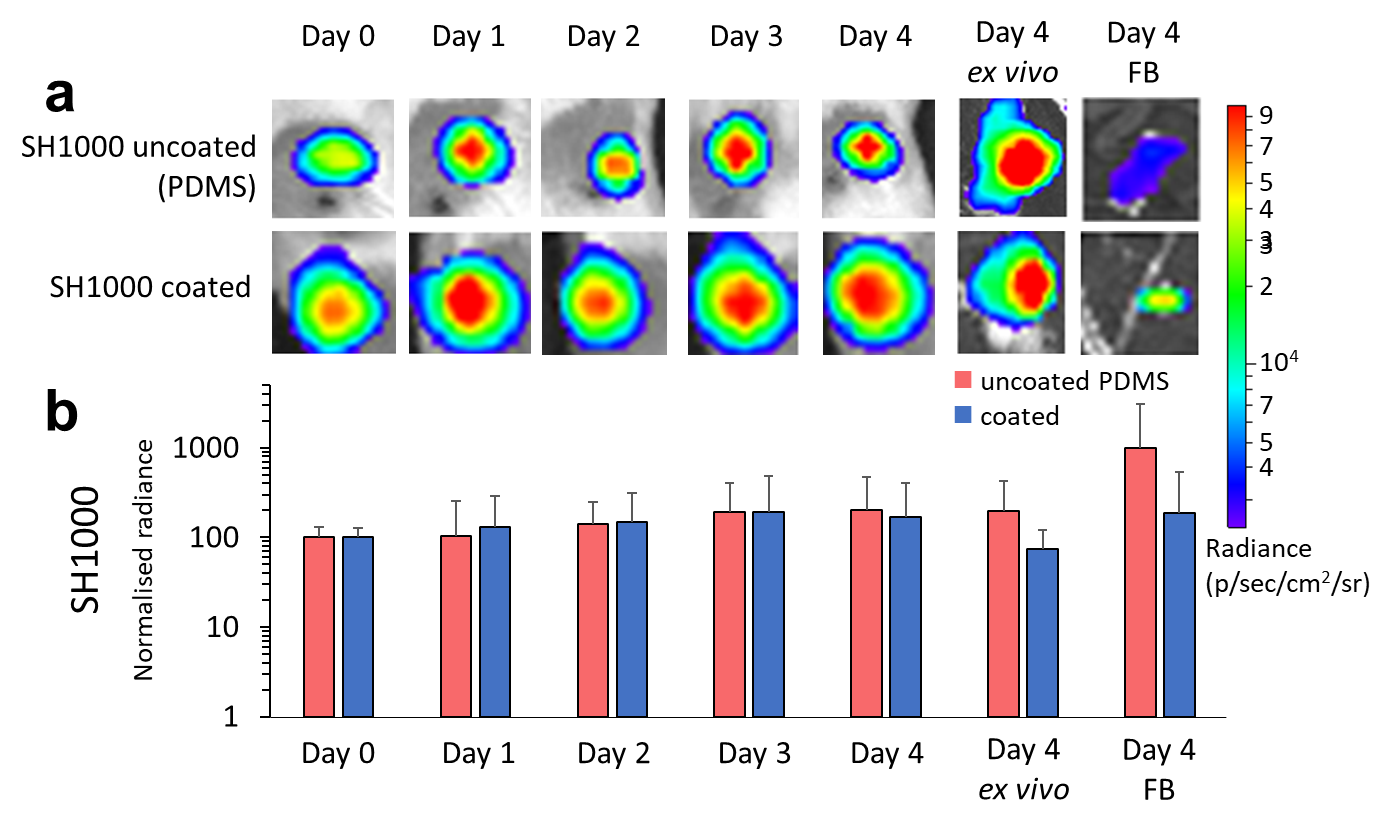


**Figure SI17.** Murine foreign body (FB) infection with *S. aureus* for testing the *in vivo* performance of the ‘hit’ copolymer. a) Luminescent images of the implantation site in live mice over 4 days for uncoated and iBnA-co-triEGMA (75:25) polymer coated silicone catheter segments inoculated with bioluminescent *S. aureus*. The FBs were implanted subcutaneously. Light output from bacteria colonizing the implanted co-polymer coated segments in whole live mice was measured on days 0 to 4. After the mice were euthanized, the catheter segments were removed and both the surrounding tissues (day 4, *ex vivo*) and the implants (day 4, FB) imaged *ex vivo*. Inset, intensity scale (radiance) where red and blue refer to high and low light outputs respectively. Image dimensions = 16 × 16 mm. b) Quantification of light output (normalized radiance) from uncoated silicone (red) and poly(iBnA-co-triEGMA) polymer coated catheter segments (blue) for *S. aureus*. Error bars show one standard deviation unit, N=8.


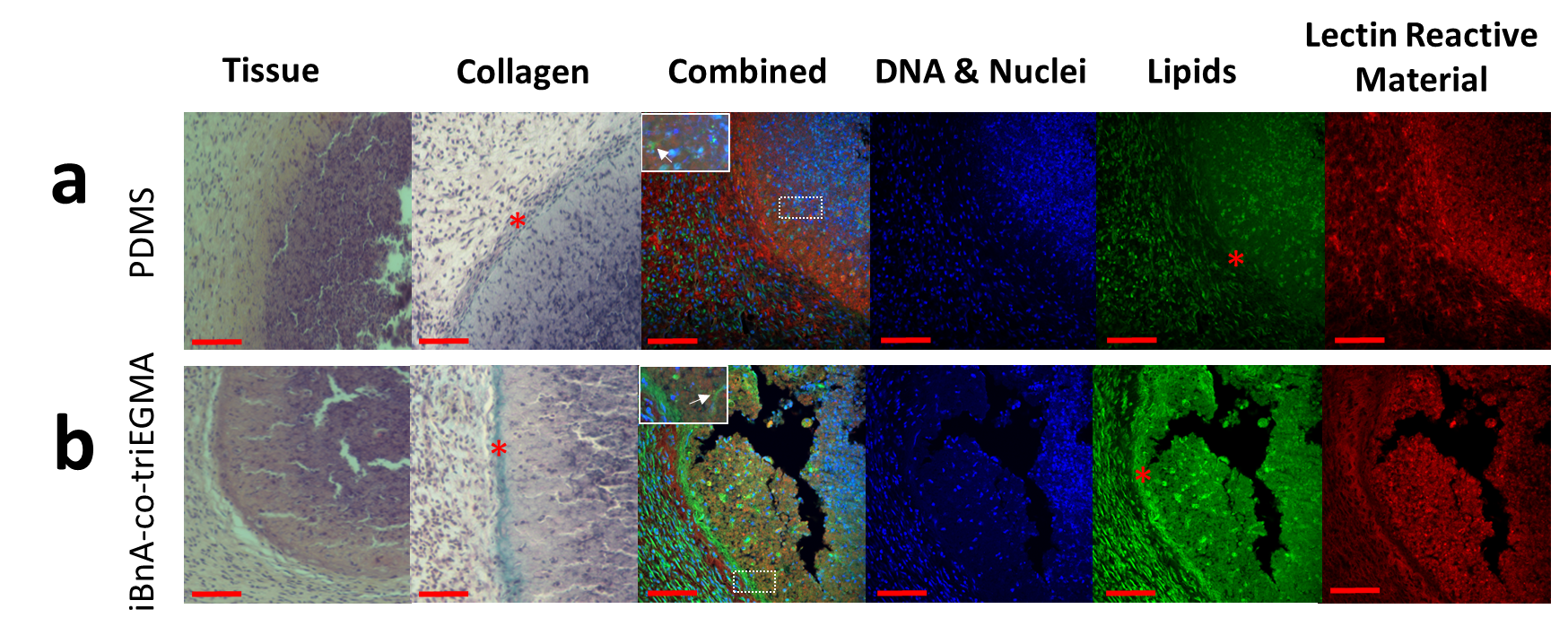
**Figure S18.** Histological analysis of tissue sections surrounding a) subcutaneously implanted silicone and b) poly (iBnA-co-triEGMA) catheter segments recovered from mice infected with *S. aureus.*  Tissue sections were stained from left to right with haematoxylin and eosin (general tissue morphology), Masson’s trichome (collagen*), combined (all 5 stains), DNA (DAPI), lipids (FM1-163) and lectins (wheat germ lectin-Alexa 680 conjugate). Of particular note is the high level of lectin reactive staining (red) in the far righthand panels indicative of a strong cellular immune response due to the presence of bacteria. The insets in the ‘combined’ panel images show localised bacterial foci (see white arrows) for both coated and uncoated implants. Scale bar equals 50 μm.
